# Gender differences in use of cigarette and non-cigarette tobacco products among adolescents aged 13–15 years in 20 African countries

**DOI:** 10.18332/tid/169753

**Published:** 2024-01-22

**Authors:** Israel T. Agaku, Rose Sulentic, Adriana Dragicevic, Gibril Njie, Candace K. Jones, Satomi Odani, Tina Tsafa, Joy Gwar, Constantine I. Vardavas, Olalekan Ayo-Yusuf

**Affiliations:** 1 School of Health Systems and Public Health, University of Pretoria, Pretoria, South Africa; 2 Centers for Disease Control and Prevention Foundation, Atlanta, United States; 3 Office on Smoking and Health, National Center for Chronic Disease Prevention and Health Promotion, Centers for Disease Control and Prevention, Atlanta, United States; 4 Osaka International Cancer Institute, Cancer Control Center, Osaka, Japan; 5 Department of Mass Communication, Benue State University, Makurdi, Nigeria; 6 Department of Clinical Psychology, Federal Medical Centre Makurdi, Makurdi, Nigeria; 7 Department of Oral Health Policy and Epidemiology, Harvard School of Dental Medicine, Boston, United States

**Keywords:** tobacco, cigarettes, policy, novel tobacco products, e-cigarettes

## Abstract

**INTRODUCTION:**

Examining gender differences in youth tobacco use is important as it aligns tobacco control within the context of broader human development goals seeking to eliminate gender inequalities. In this study, we examined gender differences in adolescent use of cigarettes, smokeless tobacco, shisha, and e-cigarettes in Africa.

**METHODS:**

This was a cross-sectional study using data from the Global Youth Tobacco Survey. Our analytical sample comprised 56442 adolescents aged 13–15 years from 20 African countries. Weighted, country-specific prevalence estimates were computed overall and by gender. Adjusted prevalence ratios (APRs) were calculated in a multivariable Poisson regression model to examine whether correlates of tobacco use differed between boys and girls.

**RESULTS:**

Ever cigarette smoking prevalence was significantly higher among boys than girls in 16 of the 20 countries, but a significantly higher percentage of girls reported earlier age of cigarette smoking initiation than boys within pooled analysis. Some of the largest gender differences in current cigarette smoking were seen in Algeria (12.2% vs 0.8%, boys and girls, respectively), Mauritius (21.2% vs 6.6%), and Madagascar (15.0% vs 4.1%). Current use of e-cigarettes, shisha, and smokeless tobacco was generally comparable between boys and girls where data existed. Among girls, higher levels of reported exposure to tobacco advertisement were positively associated with shisha smoking whereas perceived tobacco harm was inversely associated with current cigarette and shisha smoking. Among boys, perceived social acceptability of smoking at parties was associated with an increased likelihood of cigarette smoking (APR=2.27; 95% Cl: 1.20–4.30).

**CONCLUSIONS:**

The prevalence of cigarette smoking among boys was higher than that of girls in many countries. However, girls who smoke tend to start at an earlier age than boys. Differential gender patterns of cigarette and non-cigarette tobacco product use among youth may have implications for future disease burden. As the tobacco control landscape evolves, tobacco prevention efforts should focus on all tobacco products, not just cigarettes.

## INTRODUCTION

With a median age of 18.7 years, Sub-Saharan Africa is home to the world’s youngest population. The age midpoint for every other region is one or two decades older, including South-East Asia (30.0 years), Latin America and the Caribbean (30.9 years), Northern America (38.6 years), and Europe (42.7 years) ^1^. The population segment <15 years in Sub-Saharan Africa (460 million) is numerically almost seven times higher than in Northern America (67 million) and almost four times higher than in Europe (120 million) ^1^. This youthful demographic, coupled with declining cigarette sales in developed nations ^2^, has made Sub-Saharan Africa a strategic market for multinational tobacco companies ^3,4^.

Tobacco industry promotional activities in the Sub-Sahara African region^5-7^ have created a richly enabling environment for tobacco use characterized by unfettered access to cigarettes sold in single sticks, some of the cheapest cigarettes on the globe, a relatively weak tobacco regulatory climate, and aggressive marketing towards youth^8–10^.

According to recent surveys ^11–14^, cigarette smoking remains the most common form of tobacco use, with a prevalence of 10.9% of current cigarette use among adolescents in 22 African countries between 2013 and 2018^11^. The prevalence of cigarette use was higher among males (15.2%) compared to females (6.5%), and Zimbabwe had the highest prevalence of current cigarette use among adolescents (37.9%), while Morocco had the lowest (5.6%). Smokeless tobacco use was found to be the highest in Djibouti (7.6%), with a higher prevalence among males (9.7%) than females (4.9%) ^11^. Shisha smoking is also becoming increasingly common among African adolescents. For instance, 14.7% of adolescents aged 12–13 years in The Gambia reported ever smoking shisha ^13^, and in Tunisia, the prevalence of current shisha use was almost 7.2%, with higher rates among boys (13%) than girls (2.8%) ^14^. Similarly, shisha use was reported in Ghana, with 3.1% of adolescents reporting ever using a shisha in 2017 ^15^. Notably, the prevalence of current waterpipe use was higher among Ghanaian females (2.1%) than males (0.9%). Although data on e-cigarette use among African adolescents is limited, recent studies have reported concerning rates of e-cigarette use in Ghana ^15^. Specifically, a prevalence rate of 4.9% for current e-cigarette use with no differences between males and females was reported, raising the need for more research on the use of e-cigarettes among adolescents in other parts of Africa.

Previous studies have highlighted various factors influencing current tobacco use among both males and females. These include exposure to secondhand smoke (SHS) and a lack of knowledge about the harmful effects of SHS ^11,12^. Cigarette smoking was associated with exposure to tobacco industry promotions, a lack of support for smoking bans in enclosed spaces, and a lack of exposure to anti-smoking media messages^11^. Smokeless tobacco use was linked to exposure to secondhand smoke among males and exposure to tobacco industry promotions among both males and females^11^. Furthermore, shisha smoking was associated with several factors including peer influence, tobacco and drug use, and socioeconomic status ^15^.

Recent surveys within the region also show that many youths reported that a tobacco company representative had ever offered them a tobacco product, including in Zimbabwe (20.3%), Mauritania (17.8%), and Senegal (12.0%)^14^. High prevalence of youth exposure to pro-tobacco advertisements has also been documented^5,15,16^. Historically, presently, and persistently, many of these advertisements have been gender-tailored, including those explicitly targeting girls and young women with sexualized or romanticized themes^17^. It is therefore important to examine gender differences in youth receptivity to such pro-tobacco influences using indicators of tobacco experimentation and use.

Examining gender differences in youth tobacco use is further important as it aligns tobacco control within the context of broader human development goals seeking to eliminate gender inequalities^18^. Gender disparities are well documented for other public health problems in Africa, including HIV/AIDS and child illiteracy^19^. There is, however, a paucity of data on the African continent regarding gender differences in youth tobacco use and what new trends might be emerging. The extant data are over a decade old and preceded the emergence of newer tobacco products^20,21^; evaluating gender differences across the full spectrum of tobacco products is important to help modernize policies. Therefore, the objective of this study was to investigate gender differences in prevalence and patterns of use of manufactured cigarettes, smokeless tobacco, shisha, and electronic cigarettes (e-cigarettes) among African adolescents aged 13–15 years. Our secondary objective was to explore whether associated factors for tobacco use differed between boys and girls. Understanding the unique contextual factors that impact tobacco use in the different genders can help inform tailored interventions.

## METHODS

### Data source

We analyzed publicly available, de-identified secondary data from the Global Youth Tobacco Survey (GYTS) – a standardized, anonymous, probabilistic, self-administered, school-based, and nationally representative survey^16^. The survey universe comprises students within classes/grade levels coinciding with the target age of 13–15 years. Sampling is probabilistic and done in two stages. In the first stage, schools are selected with probability proportional to their enrollment size. In the second stage, classes are selected coinciding with the target age of 13–15 years; all students in each selected class are eligible to participate. GYTS has enjoyed historically high response rates; an evaluation of the surveillance system showed a median overall response rate of 85.6% (range: 57.0–97.5) among participants from 29 sites in Africa that were surveyed in the past two decades ^16^.

**Figure 1:**
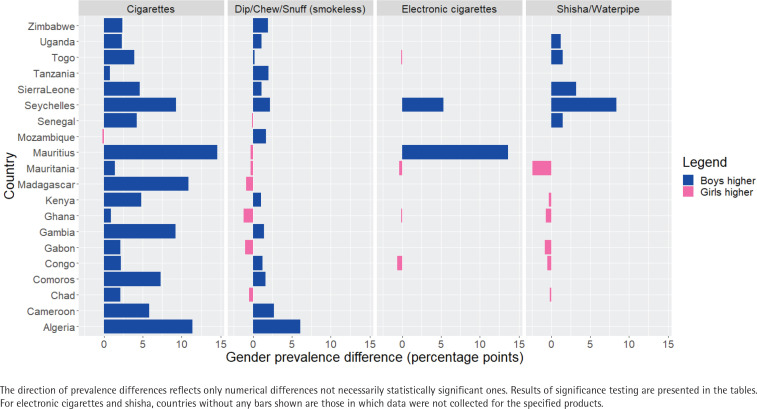
Gender difference in unadjusted prevalence of current use of different tobacco products among students aged 13–15 years in 20 countries in the WHO Africa Region, Global Youth Tobacco Survey, 2013–2020

We analyzed data for all 20 African countries located in the World Health Organization Africa (WHO AFRO) Region ^17^, whose most recent survey was fielded between 2013 and 2020 (Supplementary file Figure 1). The analytical age sample of 13–15 years combined was 56442; country-specific samples were Algeria (n=4023), Cameroon (n=1873), Chad (n=929), Comoros (n=1551), Congo (n=3672), Gabon (n=788), Gambia (n=7176), Ghana (n=5116), Kenya (n=1326), Madagascar (n=1674), Mauritania (n=2941), Mauritius (n=3076), Mozambique (n=3062), Senegal (n=2524), Seychelles (n=1525), Sierra Leone (n=3273), Tanzania (n=2527), Togo (n=2204), Uganda (n=2068), and Zimbabwe (n=5114). All 20 countries collected data on cigarettes and smokeless tobacco, 11 on shisha, and six on e-cigarettes (Figure 1). Five countries (Congo, Ghana, Mauritania, Seychelles, and Togo, total n=15458) had data for all four products.

### Key measures

*Tobacco product use*

Ever (≥1 time use in lifetime) and current use (≥1 time use in the past 30 days) were measured for all products. Participants reporting ever but not current use were classified as former users.

To compare aspects of smoking behavior among the genders beyond ever and current use prevalence, we also assessed indicators of smoking duration and intensity among users (data available only for cigarettes). Among ever cigarette smokers, age at initiation was assessed as follows: ‘How old were you when you first tried a cigarette?’. Number of cigarettes smoked per day among current cigarette smokers was assessed as follows: ‘Please think about the days you smoked cigarettes during the past 30 days. How many cigarettes did you usually smoke per day?’. Frequent cigarette smokers were those who smoked for ≥20 days.

*Indicators of pro-tobacco social influences*

We were interested in exploring the role of parents/guardians, peers, teachers, and tobacco advertisements on gender-specific tobacco use behaviors. As some of these constructs were not directly measured, we used proxy indicators. As a surrogate indicator for living with a tobacco user, we used exposure to home secondhand smoke (SHS). Participants were asked: ‘During the past 7 days, on how many days has anyone smoked inside your home, in your presence?’. Responses were dichotomized as 0 days versus ≥1 day. Similarly, exposure to SHS at school was used as a proxy for influence from peers and school personnel. Participants were asked: ‘During the past 30 days, did you see anyone smoke inside the school building or outside on school property?’ (‘Yes’ or ‘No’).

To provide a summary measure for aggregate exposure to pro-tobacco marketing, we considered the following four settings: participants saw ‘any advertisements or promotions for tobacco products at points of sale (such as kiosks, stores, shops, shopping mall, supermarket)’ in the past 30 days; they saw ‘any people using tobacco on TV, in videos, or in movies’ in the past 30 days; they owned a tobacco-branded item, and they reported that someone ‘working for a tobacco company ever offered [them] a free tobacco product’. In each setting, participants were dichotomized as exposed (‘Yes’) or non-exposed (‘No’ or reported non-contact with the assessed environment during the window period). Using these data, we created a tally for the number of settings in which exposure was reported (0, 1, 2, or ≥3).

*Perceived social acceptability of tobacco use, perceived harm, and education about tobacco harm*

Many African societies are male-dominant with distinct patterns of gender socialization ^18,19^. The perceived ethnographic significance of smoking at social gatherings may therefore differ between boys and girls. To test this empirically, we examined perceived social acceptability of smoking. Participants were asked: ‘Do you think smoking tobacco helps people feel more comfortable or less comfortable at celebrations, parties, or in other social gatherings?’. The responses were dichotomized as 0 (responses of ‘less comfortable’ or ‘no difference whether smoking or not’) or 1 (response of ‘more comfortable’).

Perceived harmfulness of tobacco smoke was assessed by: ‘Do you think the smoke from other people’s tobacco smoking is harmful to you?’. The responses were dichotomized as 0 (responses of ‘definitely not’, ‘probably not’, or ‘probably yes’) or 1 (response of ‘definitely yes’). The survey further assessed whether participants had ever seen anti-tobacco messages regarding the harmful effects of tobacco use from any source, including ‘in the media (e.g. television)’, ‘at sporting or community events’, or ‘in any of [their] classes’.

*Demographic variables*

Student demographic information included age (13, 14, and 15 years), gender (male, female), and receipt of pocket money (a proxy for disposable income, dichotomized as yes or no).

*Ecological variables*

A priori power calculations with fixed sample sizes revealed that without combining data across multiple countries, multivariable analysis would be underpowered to identify key predictors of tobacco use among boys or girls. For example, whereas a minimum of 6122 girls were needed to detect whether exposure to education about the harmfulness of tobacco use was associated with smokeless tobacco use in this subgroup (calculated based on observed prevalence of tobacco use by exposure status in the analyzed dataset), the achieved country-specific sample size for girls was at most one-third of this required sample (combined across the five countries with data for all four tobacco products, n=7922 for girls). Pooled analyses thus represented a trade-off between increased sample size (and reduced risk of type 2 statistical error) on the one hand, but increased demographic heterogeneity that must be addressed beyond a simple country-level adjustment, on the other hand. Consequently, we included three ecological variables to provide additional adjustment within the context of pooled multivariable analyses. These variables were: human development index (a composite index of life expectancy, education, and per capita income) ^20^, the percentage of the population living in urban areas (likely related to type of tobacco products used, tobacco cultivation, access to emerging tobacco products, socioeconomic status, and health literacy) ^21^, and indicators of country achievements for selected MPOWER measures (comprehensiveness of tobacco control and prevention policies) ^22^. Using the MPOWER framework (Monitoring of tobacco use; Protecting non-smokers from secondhand smoke; Offering help to quit tobacco use; Warning about the dangers of tobacco use; Enforcing restrictions on tobacco marketing; and Raising tobacco taxes), we selected six policy-related indicators, each measured on a scale of 1–4, and summed them for each country. The selected indicators were: policies on smoke-free environments (from none, or up to two public places completely smoke-free; to all public places completely smoke-free or ≥90% of the population covered by complete subnational smoke-free legislation); treatment of tobacco dependence (from none, to national quitline, and both nicotine replacement therapy and some cessation services cost-covered); health warnings on cigarette packages (from no warnings or small warnings; to large warnings with all appropriate characteristics); anti-tobacco campaigns lasting ≥3 weeks (from none during the period assessed, to national campaign conducted and aired on television or radio); bans on advertising, promotion and sponsorship (from none, or ban that does not cover national television, radio and print media, to ban on all forms of direct and indirect advertising, or ≥90% of the population protected by subnational legislation), and share of total taxes in the retail price of the most widely sold brand of cigarettes (from ≤25%, to >75% of retail price is tax) ^22^.

### Data analysis

Data were weighted to yield nationally representative estimates. Gender-stratified prevalence of ever and current use was country-specific, whereas comparative analysis of smoking frequency, duration, and intensity was performed with pooled data from all 20 countries to increase sample size. The percentage of ever users that no longer reported current use was also calculated for each tobacco product. Prevalence estimates with relative standard errors ≥30% were deemed imprecise. Two-tailed chi-squared tests were used to examine whether any observed gender differences were statistically significant (p<0.05). Only estimates measured with precision for both genders were tested.

To explore whether associated factors for current use differed between boys and girls for the different tobacco products, we used gender- and tobacco product-specific multivariable Poisson regression models to calculate adjusted prevalence ratios (APRs) for various independent variables utilizing pooled data from the five countries with information for all four tobacco products (n=15458). The two methodological issues associated with pooled analysis were addressed appropriately. The first issue, within-country clustering, was addressed by nesting the original strata variable within the country indicator and incorporating both as sources of variance within the complex survey analysis. The second issue, cross-country heterogeneity in economic, policy, and social characteristics was addressed by controlling for human development index; the percentage of the population living in urban areas (modelled in incremental percentage point increases); and the extent of tobacco prevention and control policies. In addition to these ecological variables, the other variables in the final exploratory analysis were exposures to SHS at home, SHS at school, pro-tobacco marketing, and anti-tobacco education; perceived tobacco harm; perceived social acceptability of smoking; pocket money, and survey year. Eight models were fitted (four tobacco products, stratified by boys and girls). To reduce the risk of type 1 error (false positives) from multiplicity, confidence intervals for the ensuing APRs were calculated conservatively at the 99% level. All tests were two-tailed, and all analyses were performed with R Version 3.6.3 using the ‘survey’ package.

## RESULTS

### Differences in prevalence and patterns of tobacco use by gender

The percentage of boys among the study population of participants aged 13–15 years ranged from 43.3% (Gambia) to 60.2% (Chad) (Table 1). Figure 1 shows the gender differences in current cigarette smoking prevalence compared to other tobacco products. In 16 countries for ever cigarette smoking, and in 10 for current cigarette smoking, boys reported significantly higher prevalence than girls; differences in other countries were non-significant (Table 2). The largest gender differences in current cigarette smoking were seen in Algeria (12.2% vs 0.8%, boys and girls, respectively), Mauritius (21.2% vs 6.6%), and Madagascar (15.0% vs 4.1%).

**Table 1:** Characteristics of the study population of students sampled in the Global Youth Tobacco Survey in 20 countries in the WHO Africa Region during 2013–2020

*Country*	*Survey year*	*Total sample size (all ages)^¶^* *n*	Analytical sample size for indicated population (adolescents aged 13–15 years)* *n*	*Proportion of males among the indicated population* *%*
Algeria	2013	6228	4023	43.8
Cameroon	2014	2922	1873	54.4
Chad^†^	2019	2296	929	60.2
Comoros	2015	2810	1551	45.6
Congo^§†^	2019	6396	3672	49.8
Gabon^†^	2014	1781	788	46.9
Gambia	2017	12585	7176	43.3
Ghana^§†^	2017	5664	5116	50.5
Kenya^†^	2013	1895	1326	49.5
Madagascar	2018	2920	1674	45.2
Mauritania^§†^	2018	3740	2941	50.7
Mauritius^§^	2016	4141	3076	49.0
Mozambique	2013	5599	3062	47.2
Senegal^†^	2020	4320	2524	45.7
Seychelles^§†^	2015	2485	1525	49.6
Sierra Leone^†^	2017	6680	3273	48.4
Tanzania	2016	3840	2527	48.0
Togo^§†^	2019	3917	2204	53.9
Uganda^†^	2018	3458	2068	47.8
Zimbabwe	2014	6427	5114	49.3

Percentages are weighted whereas counts (n) are unweighted. All 20 countries collected information on both cigarettes and smokeless tobacco.

§ Also collected data on e-cigarettes.

† Also collected data on shisha.

¶ A two-stage cluster sampling procedure was used to generate a representative sample. In the first stage, schools were chosen with selection probability proportional to their enrollment sizes. In the second stage, eligible classes (those coinciding with the target age group of 13–15 years) were randomly chosen and all students in the selected classes surveyed regardless of their actual age. Consequently, some of the sampled students may be younger than 13 or older than 15 years.

* Restriction of the analytical sample to participants aged 13–15 years was done to reduce confounding bias (e.g. older adolescents >15 years and preteens <13 years may both differ systematically from younger adolescents aged 13–15 years). Also, restriction of the sample allowed for direct cross-country comparisons regardless of the country-specific school grade system utilized during the sampling process.

**Table 2:** Overall and gender-specific prevalence of ever and current cigarette smoking among students aged 13-15 years sampled in the Global Youth Tobacco Survey in 20 countries in the WHO Africa Region during 2013–2020

*Country*	*Ever cigarette smoking prevalence*				*Current cigarette smoking prevalence*			
	*Overall % (95% CI)*	*Girls % (95% CI)*	*Boys % (95% CI)*	*Prevalence difference, percentage points^§^*	*Overall % (95% CI)*	*Girls % (95% CI)*	*Boys % (95% CI)*	*Prevalence difference, percentage points^§^*
Algeria	17.0 (15.1–18.9)	5.4 (4.1–6.7)	32.0 (27.8–36.2)	26.6^¶^	5.7 (4.6–6.8)	0.8 (0.3–1.4)^†^	12.2 (9.5–14.9)	11.4
Cameroon	16.5 (12.0–21.1)	10.5 (7.6–13.3)	21.7 (15.0–28.4)	11.2^¶^	5.7 (3.1–8.3)	2.5 (1.1–3.9)	8.3 (4.2–12.4)	5.8^¶^
Chad	10.1 (10.1–10.1)	7.1 (7.1–7.1)	11.5 (11.5–11.5)	4.4^¶^	1.9 (1.9–1.9)	0.6 (0.6–0.6)	2.7 (2.7–2.7)	2.1^¶^
Comoros	13.7 (10.0–17.4)	8.1 (4.6–11.7)	20.4 (15.3–25.5)	12.3^¶^	6.5 (4.2–8.9)	3.2 (1.6–4.8)	10.5 (6.3–14.6)	7.3^¶^
Congo	11.8 (9.0–14.5)	7.4 (5.4–9.4)	15.1 (10.6–19.6)	7.7^¶^	3.7 (2.5–4.9)	2.3 (1.5–3.1)	4.5 (2.6–6.4)	2.2^¶^
Gabon	23.1 (19.3–27.0)	20.3 (14.5–26.1)	26.2 (19.0–33.4)	5.9	5.2 (3.8–6.5)	4.0 (1.7–6.2)	6.1 (4.0–8.2)	2.1
Gambia	18.8 (16.5–21.0)	9.2 (7.3–11.1)	31.4 (27.8–34.9)	22.2^¶^	6.5 (5.3–7.6)	2.5 (1.5–3.5)	11.7 (9.5–13.9)	9.2^¶^
Ghana	8.7 (6.7–10.6)	6.1 (4.0–8.3)	11.2 (8.5–13.9)	5.1^¶^	2.8 (1.7–3.9)	2.3 (0.5–4.2)^†^	3.2 (2.3–4.1)	0.9
Kenya	13.7 (9.6–17.7)	8.8 (5.4–12.2)	18.5 (11.7–25.3)	9.7^¶^	4.9 (3.0–6.9)	2.6 (0.7–4.5)^†^	7.4 (4.0–10.7)	4.8
Madagascar	23.2 (17.8–28.7)	11.8 (7.5–16.1)	37.3 (26.8–47.8)	25.5^¶^	8.9 (6.1–11.7)	4.1 (2.0–6.2)	15.0 (9.8–20.2)	10.9^¶^
Mauritania	20.9 (17.3–24.6)	15.7 (11.5–19.9)	25.4 (20.2–30.5)	9.7 ^¶^	13.1 (8.0–18.2)	12.0 (5.0–19.0)	13.4 (9.0–17.9)	1.4
Mauritius	28.2 (22.1–34.2)	16.3 (10.7–21.9)	40.6 (35.5–45.7)	24.3^¶^	13.6 (9.1–18.1)	6.6 (3.6–9.5)	21.2 (15.6–26.8)	14.6^¶^
Mozambique	8.6 (7.0–10.2)	6.6 (4.8–8.4)	10.1 (8.0–12.2)	3.5^¶^	2.3 (1.4–3.2)	2.3 (1.1–3.5)	2.1 (0.9–3.2)	-0.2
Senegal	9.6 (7.5–11.6)	4.6 (2.9–6.3)	15.2 (12.0–18.4)	10.6^¶^	3.4 (2.4–4.5)	1.4 (0.7–2.1)	5.6 (3.3–7.8)	4.2^¶^
Seychelles	38.3 (33.9–42.7)	32.4 (26.9–37.9)	44.4 (39.6–49.2)	12^¶^	14.7 (12.0–17.5)	10.3 (7.2–13.4)	19.6 (16.0–23.2)	9.3^¶^
Sierra Leone	12.6 (8.4–16.8)	10.7 (4.2–17.3)^†^	14.8 (10.9–18.8)	4.1	3.7 (1.9–5.5)	1.6 (0.3–2.8)^†^	6.2 (3.3–9.0)	4.6
Tanzania	5.2 (3.6–6.8)	2.2 (1.1–3.4)	7.8 (5.3–10.4)	5.6^¶^	1.3 (0.7–1.8)	0.7 (0.2–1.3)^†^	1.5 (0.5–2.5)	0.8
Togo	8.9 (3.0–14.8)^†^	6.5 (0.0–15.1)^†^	11.0 (6.4–15.7)	4.5	2.8 (1.8–3.8)	0.7 (0.2–1.2)^†^	4.6 (2.8–6.4)	3.9
Uganda	16.0 (11.4–20.6)	8.7 (5.1–12.3)	23.4 (16.6–30.2)	14.7^¶^	3.5 (1.7–5.3)	2.4 (1.0–3.8)	4.7 (1.8–7.6)	2.3^¶^
Zimbabwe	18.6 (13.0–24.3)	15.8 (9.8–21.8)	20.4 (14.4–26.4)	4.6	11.2 (6.0–16.5)	8.9 (4.2–13.6)	11.3 (5.9–16.7)	2.4

§ Prevalence difference calculated as prevalence among boys minus prevalence among girls.

† Imprecise prevalence estimates (relative standard errors ≥30%).

¶ Statistically significant prevalence difference between boys and girls (p<0.05).

Of ever cigarette smokers, the percentage that no longer reported current use was significantly higher among girls than boys in many countries including Algeria (83.9% vs 59.1%), Cameroon (71.6% vs 57.6%), Chad (91.3% vs 75.0%), Comoros (60.4% vs 41.1%), Gambia (68.3% vs 57.4%), Seychelles (67.7% vs 52.3%), Sierra Leone (85.9% vs 53.1%), and Togo (89.1% vs 55.2%) (all p<0.05). Other differences were non-significant (data not shown in tables).

Among ever cigarette smokers in all 20 countries combined, a significantly higher proportion of girls reported an earlier age of initiation than boys (p=0.010). Among girls who had ever smoked cigarettes, frequency distribution of age at initiation was: <7 (23.6%), 8–9 (15.9%), 10–11 (21.6%), 12–13 (19.1%), and 14–15 years (19.8%). The corresponding distributions among boys who had ever smoked cigarettes were: <7 (17.9%), 8–9 (16.0%), 10–11 (21.3%), 12–13 (28.0%), and 14–15 years (16.8%). Among youth who currently smoked cigarettes in the pooled dataset, no significant gender differences existed in frequent smoking (p=0.4246), or cigarettes smoked per day (p=0.273) (data not shown in tables).

Of assessed countries, only few had significant gender differences in ever and current use of the assessed non-cigarette tobacco products (Tables 3–5). Gender differences were statistically significant in only four countries for current smokeless tobacco use (prevalence higher among boys in Cameroon, Gambia, Mozambique; prevalence higher among girls in Chad), three countries for current shisha smoking (prevalence higher among boys in Senegal and Seychelles; prevalence higher among girls in Chad), and one country for current e-cigarette use (prevalence higher among boys in Seychelles). These differences, while statistically significant, were smaller in magnitude relative to cigarettes.

**Table 3:** Overall and gender-specific prevalence of ever and current smokeless tobacco use among students aged 13–15 years sampled in the Global Youth Tobacco Survey in 20 countries in the World Health Organization Africa Region during 2013–2020

*Country*	*Ever smokeless tobacco use*				*Current smokeless tobacco use*			
	*Overall % (95% CI)*	*Girls % (95% CI)*	*Boys % (95% CI)*	*Prevalence difference, percentage points^§^*	*Overall % (95% CI)*	*Girls % (95% CI)*	*Boys % (95% CI)*	*Prevalence difference, percentage points^§^*
Algeria	6.6 (5.3–7.9)	1.5 (1.0–1.9)	13.1 (10.0–16.3)	11.6^¶^	3.5 (2.6–4.3)	0.8 (0.3–1.4)^†^	6.9 (5.2–8.6)	6.1
Cameroon	9.6 (6.6–12.5)	8.0 (4.8–11.2)	10.9 (6.8–15.0)	2.9	3.7 (2.2–5.3)	2.3 (0.9–3.6)	5.0 (2.9–7.2)	2.7^¶^
Chad	10.1 (10.1–10.1)	9.7 (9.7–9.7)	10.5 (10.5–10.5)	0.8^¶^	5.8 (5.8–5.8)	6.0 (6.0–6.0)	5.5 (5.5–5.5)	–0.5^¶^
Comoros	7.6 (6.3–9.0)	7.0 (4.9–9.1)	8.5 (6.3–10.7)	1.5	2.7 (2.0–3.5)	2.0 (1.0–3.1)	3.6 (1.9–5.2)	1.6
Congo	13.1 (10.4–15.9)	11.3 (9.0–13.7)	14.4 (10.8–17.9)	3.1^¶^	7.5 (5.9–9.0)	6.7 (4.9–8.4)	7.9 (5.7–10.0)	1.2
Gabon	6.4 (4.3–8.5)	7.0 (4.0–10.1)	5.4 (2.8–8.0)	–1.6	2.4 (1.8–3.1)	2.9 (1.3–4.5)	1.9 (0.7–3.1)	-1.0
Gambia	4.6 (3.9–5.3)	3.5 (2.8–4.2)	5.8 (4.7–7.0)	2.3^¶^	1.5 (1.2–1.9)	0.9 (0.6–1.2)	2.3 (1.6–3.0)	1.4^¶^
Ghana	9.2 (6.9–11.4)	8.3 (5.7–10.9)	9.4 (7.0–11.8)	1.1	3.1 (2.1–4.2)	3.7 (1.9–5.5)	2.5 (1.8–3.3)	-1.2
Kenya	10.9 (9.0–12.8)	8.8 (6.8–10.8)	12.9 (9.8–16.0)	4.1^¶^	3.9 (2.8–5.0)	3.3 (2.1–4.6)	4.3 (2.4–6.2)	1.0
Madagascar	8.4 (4.9–12.0)	9.2 (4.1–14.2)	7.6 (3.8–11.3)	–1.6	1.6 (0.6–2.5)^†^	2.0 (0.3–3.8)^†^	1.1 (0.1–2.0)	-0.9
Mauritania	12.6 (10.5–14.7)	12.4 (9.5–15.3)	12.5 (9.7–15.2)	0.1	6.8 (5.7–7.9)	6.8 (4.9–8.7)	6.5 (4.7–8.4)	-0.3
Mauritius	5.2 (3.6–6.7)	4.3 (2.3–6.4)	6.0 (4.1–8.0)	1.7	2.3 (1.5–3.1)	2.4 (1.1–3.8)	2.1 (1.2–3.1)	-0.3
Mozambique	8.8 (7.3–10.4)	7.9 (5.9–9.8)	9.4 (6.8–12.0)	1.5	4.3 (3.3–5.2)	3.3 (2.3–4.4)	5.0 (3.5–6.5)	1.7^¶^
Senegal	6.9 (4.3–9.5)	5.7 (3.7–7.7)	8.2 (4.6–11.8)	2.5^¶^	3.5 (2.0–5.1)	3.5 (2.1–5.0)	3.4 (1.2–5.7)	-0.1
Seychelles	4.9 (3.6–6.1)	3.3 (2.0–4.6)	6.5 (4.3–8.7)	3.2^¶^	1.7 (1.0–2.4)	0.6 (0.1–1.2)^†^	2.8 (1.5–4.1)	2.2
Sierra Leone	12.1 (7.9–16.3)	9.9 (5.6–14.2)	13.7 (8.1–19.3)	3.8	6.0 (2.7–9.3)	5.4 (1.8–9.1)^†^	6.5 (2.7–10.2)	1.1
Tanzania	4.7 (3.3–6.1)	2.7 (1.1–4.3)	6.1 (4.4–7.8)	3.4^¶^	2.1 (1.2–3.0)	0.9 (0.2–1.7)^†^	2.9 (1.4–4.3)	2.0
Togo	3.3 (1.9–4.7)	2.2 (0.8–3.6)^†^	4.1 (2.1–6.1)	1.9	0.7 (0.3–1.1)^†^	0.6 (0.0–1.2)^†^	0.8 (0.1–1.4)	0.2
Uganda	11.8 (8.4–15.1)	11.0 (6.0–15.9)	12.6 (8.9–16.3)	1.6	6.5 (4.0–9.0)	6.0 (1.5–10.5)^†^	7.1 (4.3–9.8)	1.1
Zimbabwe	15.7 (10.7–20.6)	14.6 (9.6–19.6)	16.2 (10.9–21.6)	1.6	5.6 (4.0–7.2)	4.6 (2.8–6.4)	6.5 (4.4–8.5)	1.9

§ Prevalence difference calculated as prevalence among boys minus prevalence among girls.

† Imprecise prevalence estimates (relative standard errors ≥30%).

¶ Statistically significant prevalence difference between boys and girls (p<0.05).

**Table 4:** Overall and gender-specific prevalence of ever and current use of shisha among students aged 13–15 years sampled in the Global Youth Tobacco Survey in 11 countries in the WHO Africa Region that collected data on shisha during 2013–2020

*Country*	*Ever use*				*Current use*			
	*Overall % (95% CI)*	*Girls % (95% CI)*	*Boys % (95% CI)*	*Prevalence difference, percentage points^§^*	*Overall % (95% CI)*	*Girls % (95% CI)*	*Boys % (95% CI)*	*Prevalence difference, percentage points^§^*
Chad	17.2 (17.2–17.2)	15.5 (15.5–15.5)	17.9 (17.9–17.9)	2.4^¶^	0.7 (0.7–0.7)	0.8 (0.8–0.8)	0.6 (0.6–0.6)	-0.2^¶^
Congo	12.9 (10.6–15.2)	12.4 (9.6–15.3)	12.6 (9.9–15.2)	0.2	2.9 (1.9–3.9)	2.8 (1.5–4.0)	2.3 (1.5–3.0)	-0.5
Gabon	6.2 (4.2–8.3)	5.0 (1.8–8.3)	7.5 (5.4–9.6)	2.5	2.4 (1.2–3.7)	2.8 (1.1–4.5)	2.0 (0.0–5.0)^†^	-0.8
Ghana	8.8 (6.4–11.2)	7.8 (5.0–10.6)	9.2 (6.4–12.0)	1.4	5.3 (3.3–7.2)	5.4 (2.5–8.3)	4.7 (2.9–6.5)	-0.7
Kenya	6.5 (4.6–8.5)	4.8 (3.0–6.7)	7.7 (4.8–10.7)	2.9^¶^	5.4 (3.4–7.3)	5.4 (3.1–7.6)	5.1 (2.2–7.9)	-0.3
Mauritania	18.1 (14.3–21.8)	16.7 (11.5–22.0)	19.0 (15.1–22.8)	2.3	18.2 (12.4–23.9)	19.1 (10.9–27.2)	16.7 (12.2–21.1)	-2.4
Senegal	9.8 (7.5–12.0)	6.9 (4.8–9.0)	13.2 (10.1–16.2)	6.3^¶^	2.2 (1.4–2.9)	1.4 (0.8–2.0)	2.9 (1.4–4.4)	1.5^¶^
Seychelles	25.1 (21.8–28.3)	19.6 (15.8–23.3)	30.7 (26.4–35.0)	11.1^¶^	13.8 (11.1–16.4)	9.6 (6.8–12.4)	18.0 (14.2–21.8)	8.4^¶^
Sierra Leone	14.0 (10.6–17.5)	12.1 (8.8–15.3)	16.0 (11.2–20.8)	3.9^¶^	6.5 (3.3–9.6)	4.7 (1.7–7.7)^†^	7.9 (3.8–12.1)	3.2
Togo	4.9 (3.3–6.4)	2.5 (1.4–3.7)	7.0 (4.4–9.5)	4.5^¶^	1.1 (0.6–1.6)	0.3 (0.0–0.6)^†^	1.8 (0.8–2.7)	1.5
Uganda	5.7 (3.9–7.5)	4.7 (2.6–6.7)	7.0 (5.1–8.9)	2.3^¶^	1.7 (0.8–2.5)	1.1 (0.1–2.1)^†^	2.3 (0.6–4.0)^†^	1.2

§ Prevalence difference calculated as prevalence among boys minus prevalence among girls.

† Imprecise prevalence estimates (relative standard errors ≥30%).

¶ Statistically significant prevalence difference between boys and girls (p<0.05).

*Data not available.

**Table 5:** Overall and gender-specific prevalence of ever and current use of e-cigarettes among students aged 13–15 years sampled in the Global Youth Tobacco Survey in six countries in the WHO Africa Region that collected data on e-cigarettes during 2013–2020

*Country*	*Ever use*				*Current use*			
	*Overall % (95% CI)*	*Girls % (95% CI)*	*Boys % (95% CI)*	*Prevalence difference, percentage points^§^*	*Overall % (95% CI)*	*Girls % (95% CI)*	*Boys % (95% CI)*	*Prevalence difference, percentage points^§^*
Congo	9.7 (6.6–12.9)	8.4 (5.7–11.0)	8.5 (6.0–11.0)	0.1	6.0 (3.1–8.8)	5.1 (3.2–7.0)	4.5 (2.9–6.0)	-0.6
Ghana	7.9 (5.2–10.7)	7.8 (4.3–11.2)	7.5 (4.6–10.4)	-0.3	4.9 (3.0–6.9)	5.0 (2.5–7.5)	4.9 (2.4–7.4)	-0.1
Mauritania	NA	NA	NA		18.8 (13.4–24.1)	18.5 (11.3–25.6)	18.1 (12.7–23.5)	-0.4
Mauritius	NA	NA	NA		10.9 (7.6–14.3)	4.3 (1.6–6.9)^†^	17.9 (14.4–21.3)	13.6
Seychelles	11.2 (8.8–13.7)	7.8 (4.8–10.9)	14.7 (11.6–17.7)	6.9^¶^	7.3 (5.4–9.3)	4.7 (2.8–6.6)	10.0 (7.1–12.9)	5.3^¶^
Togo	NA	NA	NA		1.0 (0.5–1.6)	1.1 (0.2–2.1)^†^	1.0 (0.5–1.5)	-0.1

§ Prevalence difference calculated as prevalence among boys minus prevalence among girls.

† Imprecise prevalence estimates (relative standard errors ≥30%).

¶ Statistically significant prevalence difference between boys and girls (p<0.05).

NA: data not available.

### Differences in associated factors for current tobacco use by gender

Common risk factors for tobacco use were observed, although the strength of association differed by gender (Table 6). Home (but not school) SHS exposure among girls was significantly associated with higher probability of currently using cigarettes (APR=3.39; 99% Cl: 1.87–6.13), smokeless tobacco (APR=2.12; 95% CI: 1.04–4.32), and e-cigarettes (APR=2.59; 95% CI: 1.38–4.85). Among boys, home SHS was also associated with current use of cigarettes (APR=1.96; 95% CI: 1.01–3.83) and e-cigarettes (APR=5.28; 95% CI: 1.57–17.72). The perception of tobacco smoke as ‘definitely harmful’ was inversely associated with current smoking of shisha (APR=0.50; 95% CI: 0.27–0.92) among girls as well as current use of e-cigarettes among boys (APR=0.42; 95% CI: 0.20–0.87). Perceived social acceptability of smoking at parties was associated with increased probability of currently smoking cigarettes among boys (APR=2.27; 95% Cl: 1.20–4.30) but non-significant among girls (Table 6).

**Table 6:** Factors associated with current use of cigarettes, shisha, e-cigarettes, and smokeless tobacco products among students aged 13–15 years sampled in the Global Youth Tobacco Survey in five countries^§^ in the WHO Africa Region that collected data on all four assessed tobacco products during 2015–2019 (N=15458)

*Outcome variable (current use)^†^*	*Predictor variables*	*Categories of predictor variable*	*Associations among girls APR (95% CI)*	*Associations among boys APR (95% CI)*
**Cigarettes**	Home secondhand smoke exposure reported	Yes vs No	3.39 (1.87–6.13)^¶^	1.96 (1.01–3.83)^¶^
	School secondhand smoke exposure reported	Yes vs No	1.50 (0.95–2.38)	1.35 (0.79–2.29)
	Number of settings in which exposed to tobacco marketing	One vs none	2.94 (1.08–7.98)^¶^	1.50 (0.79–2.87)
		Two vs none	5.55 (2.08–14.84)^¶^	2.66 (1.48–4.77)^¶^
		≥Three vs none	5.73 (1.96–16.74)^¶^	3.76 (1.38–10.26)^¶^
	Perceive tobacco smoke as ‘definitely harmful’	Yes vs No	0.43 (0.18–1.00)	0.63 (0.38–1.07)
	Perceive that smoking at parties makes one comfortable	Yes vs No	1.13 (0.55–2.34)	2.27 (1.20–4.30)^¶^
	Survey year	Per unit increase	1.45 (0.69–3.04)	1.80 (1.19–2.73)^¶^
	Received any education about the dangers of tobacco use	Yes vs No	0.79 (0.34–1.80)	0.94 (0.52–1.69)
	Current shisha use reported	Yes vs No	5.09 (2.65–9.78)^¶^	2.49 (1.33–4.65)^¶^
	Current smokeless tobacco use reported	Yes vs No	2.02 (1.03–3.94)^¶^	1.94 (0.98–3.83)
	Current e-cigarette use reported	Yes vs No	3.24 (1.36–7.73)^¶^	2.14 (1.19–3.83)^¶^
	Reported pocket money	Yes vs No	1.85 (0.96–3.57)	1.47 (0.79–2.75)
	Urbanicity	Per unit increase	0.99 (0.93–1.05)	0.93 (0.90–0.97)^¶^
	Tobacco control scale	Per unit increase	0.91 (0.83–0.99)^¶^	0.93 (0.88–0.98)^¶^
	Human development index	Per unit increase	1.13 (1.02–1.27)^¶^	1.15 (1.07–1.23)^¶^
**Shisha**	Home secondhand smoke exposure reported	Yes vs No	1.27 (0.64–2.52)	1.71 (0.91–3.21)
	School secondhand smoke exposure reported	Yes vs No	1.15 (0.74–1.78)	1.67 (0.89–3.14)
	Number of settings in which exposed to tobacco marketing	One vs none	1.58 (0.70–3.58)	1.45 (0.50–4.19)
		Two vs none	1.99 (0.87–4.56)	2.41 (0.84–6.88)
		≥Three vs none	3.66 (1.49–9.04)^¶^	2.40 (0.79–7.31)
	Perceive tobacco smoke as ‘definitely harmful’	Yes vs No	0.50 (0.27–0.92)^¶^	0.69 (0.34–1.40)
	Perceive that smoking at parties makes one comfortable	Yes vs No	1.28 (0.76–2.16)	1.73 (0.96–3.14)
	Survey year	Per unit increase	0.99 (0.51–1.92)	0.98 (0.57–1.67)
	Received any education about the dangers of tobacco use	Yes vs No	1.27 (0.71–2.28)	1.17 (0.68–2.03)
	Current cigarette use reported	Yes vs No	1.93 (1.02–3.65)^¶^	2.76 (1.29–5.92)^¶^
	Current smokeless tobacco use reported	Yes vs No	0.76 (0.44–1.31)	1.48 (0.70–3.10)
	Current e-cigarette use reported	Yes vs No	23.16 (10.07–53.23)^¶^	6.39 (3.57–11.44)^¶^
	Reported pocket money	Yes vs No	0.69 (0.43–1.08)	1.00 (0.61–1.62)
	Urbanicity	Per unit increase	1.03 (0.95–1.11)	0.95 (0.91–0.99)^¶^
	Tobacco control scale	Per unit increase	0.87 (0.82–0.92)^¶^	0.90 (0.84–0.97)^¶^
	Human development index	Per unit increase	1.08 (0.99–1.18)	1.07 (0.99–1.17)
**E–cigarettes**	Home secondhand smoke exposure reported	Yes vs No	2.59 (1.38-4.85)^¶^	5.28 (1.57–17.72)^¶^
	School secondhand smoke exposure reported	Yes vs No	1.22 (0.76–1.96)	1.06 (0.65–1.72)
	Number of settings in which exposed to tobacco marketing	One vs none	1.00 (0.54–1.82)	0.71 (0.25–2.03)
		Two vs none	1.54 (0.82–2.91)	1.22 (0.46–3.24)
		≥Three vs none	1.91 (0.71–5.14)	1.20 (0.37–3.91)
	Perceive tobacco smoke as ‘definitely harmful’	Yes vs No	0.55 (0.29–1.04)	0.42 (0.20–0.87)^¶^
	Perceive that smoking at parties makes one comfortable	Yes vs No	0.99 (0.63–1.56)	0.92 (0.58–1.48)
	Survey year	Per unit increase	0.80 (0.47–1.36)	0.71 (0.42–1.22)
	Received any education about the dangers of tobacco use	Yes vs No	0.90 (0.48–1.68)	0.33 (0.14–0.79)^¶^
	Current cigarette use reported	Yes vs No	1.05 (0.52–2.09)	1.91 (1.05–3.48)^¶^
	Current smokeless tobacco use reported	Yes vs No	1.59 (0.95–2.64)	2.20 (1.23–3.93)^¶^
	Current shisha use reported	Yes vs No	20.76 (10.06–42.85)^¶^	5.99 (3.32–10.83)^¶^
	Reported pocket money	Yes vs No	0.99 (0.59–1.66)	0.64 (0.38–1.09)
	Urbanicity	Per unit increase	1.01 (0.96–1.07)	1.02 (0.96–1.09)
	Tobacco control scale	Per unit increase	0.97 (0.91–1.03)	0.89 (0.84–0.94)^¶^
	Human development index	Per unit increase	0.97 (0.89–1.06)	1.01 (0.93–1.09)
**Smokeless tobacco**	Home secondhand smoke exposure reported	Yes vs No	2.12 (1.04–4.32)^¶^	1.26 (0.49–3.28)
	School secondhand smoke exposure reported	Yes vs No	1.67 (0.90–3.09)	1.26 (0.79–1.99)
	Number of settings in which exposed to tobacco marketing	One vs none	1.38 (0.58–3.26)	0.61 (0.34–1.11)
		Two vs none	1.67 (0.71–3.93)	1.32 (0.66–2.64)
		≥Three vs none	2.12 (0.81–5.57)	2.10 (0.69–6.34)
	Perceive tobacco smoke as ‘definitely harmful’	Yes vs No	1.03 (0.50–2.11)	1.08 (0.62–1.90)
	Perceive that smoking at parties makes one comfortable	Yes vs No	1.90 (0.78–4.61)	1.35 (0.73–2.49)
	Survey year	Per unit increase	0.66 (0.31–1.43)	1.02 (0.58–1.79)
	Received any education about the dangers of tobacco use	Yes vs No	1.24 (0.63–2.43)	1.63 (0.79–3.34)
	Current cigarette use reported	Yes vs No	2.47 (0.88–6.87)	2.43 (0.95–6.19)
	Current e-cigarette use reported	Yes vs No	2.62 (1.08–6.38)^¶^	2.71 (1.15–6.39)^¶^
	Current shisha use reported	Yes vs No	0.64 (0.22–1.84)	2.06 (0.90–4.70)
	Reported pocket money	Yes vs No	0.47 (0.21–1.04)	1.00 (0.59–1.69)
	Urbanicity	Per unit increase	1.13 (1.03–1.24)^¶^	1.08 (1.03–1.15)^¶^
	Tobacco control scale	Per unit increase	0.95 (0.87–1.04)	0.96 (0.89–1.03)
	Human development index	Per unit increase	0.87 (0.75–1.01)	0.96 (0.86–1.06)

APR: adjusted prevalence ratio; analysis adjusted for all predictor variables listed in the table.

§ Included countries were Congo, Ghana, Mauritania, Seychelles, and Togo. These had information on all four tobacco product types. Potential variance from country was accounted for by including country of origin as part of the strata when creating the survey object for analysis.

† Current use was defined as past-30-day use.

¶ Statistically significant results at the 95% confidence level.

In a dose-dependent manner, exposure to tobacco marketing was significantly associated with current cigarette and shisha smoking among girls but with only cigarette smoking among boys. Compared to girls who did not report exposure to tobacco marketing from any setting, higher probability of currently smoking cigarettes was seen among girls reporting exposure from one (APR=2.94; 95% CI: 1.08–7.98), two (APR=5.55; 95% CI: 2.08–14.84), or ≥3 settings (APR=5.73; 95% CI: 1.96–16.74); higher probability of shisha smoking was also seen among girls exposed to tobacco marketing from ≥3 settings (APR=3.66; 95% CI: 1.49–9.04). Similarly, the probability of currently smoking cigarettes was higher among boys reporting marketing exposure from two (APR=2.66; 95% CI: 1.48–4.77), or ≥3 settings (APR=3.76; 95% CI: 1.38–10.26), than none (Table 6). Current use of e-cigarettes was strongly associated with current shisha smoking among both genders, although the strength of association was stronger for girls (APR=23.16; 95% CI: 10.07–53.23) than boys (APR=6.39; CI: 3.57–11.44). For every unit increase in the percentage of the population that lived in urban areas, the probability of using smokeless tobacco products increased among both girls (APR=1.13; 95% CI: 1.03–1.24) and boys (APR=1.08; 95% CI: 1.03–1.15). Conversely, living in more urban areas was associated with lowered probability of smoking cigarettes (APR=0.93; 95% CI: 0.90–0.97) and shisha (APR=0.95; 95% CI: 0.91– 0.99) among boys but not girls. Report of pocket money was not associated with any form of tobacco use among either gender nor was self-reported exposure to education about the harmfulness of tobacco use among girls. Boys, however, were less likely to report current e-cigarette use if they were exposed to education about the harmfulness of tobacco use (APR=0.33; 95% CI: 0.14–0.79), or perceived tobacco smoke as ‘definitely harmful’ (APR=0.42; 95% CI: 0.20–0.87). Stronger tobacco control measures at the national level were associated with reduced probability of using cigarettes, shisha, and e-cigarettes. For every unit increase in the extent of tobacco control, probabilities for current use decreased among girls by 9% for cigarettes and 13% for shisha; for boys, probabilities decreased by 7% for cigarettes, 10% for shisha, and 11% for e-cigarettes (Table 6). Human development index of the respondent’s country was not significantly associated with use of e-cigarettes, shisha, or smokeless tobacco, but was positively associated with cigarette smoking among both boys and girls (Table 6).

## DISCUSSION

Cigarette smoking prevalence was higher among boys, but among all adolescents who had ever smoked, a higher proportion of girls had initiated cigarette smoking at an earlier age. These distinct gender patterns of smoking might have implications for future burden of disease in the region. The higher smoking prevalence among boys might result in a large absolute number of future adult male smokers if these trends continue. For girls, the earlier age of initiation may result in higher risk of tobacco-related diseases for those who continue smoking, given that duration of smoking strongly predicts risk of tobacco-related disease and death^23^. With budgetary constraints for healthcare in the region^24^ and against the backdrop of other competing public health priorities, implementing evidence-based tobacco prevention strategies may benefit public health. Adolescents are an important demographic as they influence tobacco use behaviors among preteens, while foreshadowing adult tobacco use. A multipronged prevention approach that includes absolute prevention in the pre-teen years as well as targeted prevention in teens may be a useful strategy in each of these 20 countries. Solely targeting adolescents as a priority age group for tobacco prevention may, however, be suboptimal since many ever users begin well before the age of 10 years ^25^.

Both gender-specific prevalence and gender gaps were larger for cigarettes than other products, possibly reflecting differences in accessibility and acceptability. Novel products like e-cigarettes are akin to ‘luxury goods’ and remain an urban phenomenon in the region ^7^. Despite its deep cultural roots in certain parts of Northern Africa^26^, shisha is still relatively new elsewhere on the continent, and its usage is sharply demarcated by geographical location. Locally manufactured smokeless tobacco products, while widely available, are more popular among older adults^27^ and particularly among women, because they are more discrete to use and socially acceptable unlike smoking, which is often culturally considered a taboo for women to engage in^28^. In recent years, however, newer, sleeker smokeless tobacco products have been introduced into some African countries^27^ which may explain the increased likelihood for smokeless tobacco use among those living in more urban areas. As newer tobacco products enter the market, it will be important to monitor for shifts in gender patterns of use, including transitions to other products^29^.

Some of the identified factors associated with tobacco product use differed between cigarette and non-cigarette tobacco products and between genders. For example, perceived harmfulness of tobacco smoke was associated with reduced likelihood of current shisha smoking among girls but not boys, whereas perceived harm reduced the likelihood of e-cigarette use among boys but not girls. Associations between exposure to pro-tobacco marketing and the use of cigarettes or shisha, were stronger among girls than boys. Furthermore, being exposed to smoking within the home was associated with a higher likelihood of using cigarettes and e-cigarettes for both boys and girls, whereas exposure to smoking within the school was not associated with either outcome. This suggests that family influences may play a greater role than external inter-personal influences for tobacco use at this age. Furthermore, as living with a person who smokes may facilitate easier access to tobacco products among adolescents, youth tobacco prevention campaigns should consider extending help to smoking parents/guardians to encourage quitting. Our results also suggest a need for revamped youth-oriented tobacco educational messages and curricula^30^, as we found no association between any exposure to anti-tobacco messages and many forms of tobacco use, especially among girls.

In our study, pocket money was not associated with any form of tobacco use among both males and females. This contrasts with findings of studies among Chinese and Indonesian adolescents, that show an association between pocket money and tobacco use^31,32^. There could be several reasons why our findings differ from those of previous studies. One possibility is that African teenagers are obtaining tobacco products through alternative means, such as from older individuals or by stealing, borrowing, or being offered cigarettes by tobacco companies^33^. Additionally, while previous literature has shown that most African adolescents obtain tobacco products from stores^33^, it is possible that informal markets such as street vendors or illegal channels, like smuggling or counterfeiting, could also be sources of tobacco products at low prices^34^. It would be important for future research to investigate the specific mechanisms by which African teenagers obtain tobacco products in order to better understand patterns of tobacco use and inform effective tobacco control policies and interventions.

Our study revealed that in Africa, a greater proportion of girls than boys started smoking at an earlier age. While smoking rates and the age at which individuals start smoking vary from country to country, our findings may be influenced by several factors such as parental, sibling, and peer smoking habits^35^. Moreover, our research demonstrated a correlation between exposure to pro-tobacco marketing and cigarette or shisha use in girls more than boys, which could also contribute to the higher incidence of earlier initiation among girls. Nonetheless, in other non-African countries such as the US and England, boys tend to initiate smoking at a younger age^36,37^.

There was a strong association between current use of e-cigarettes and current shisha use, especially among girls. E-shisha/e-hookah are becoming very popular among youth and are perceived to be more ‘trendy’ compared to conventional e-cigarettes^38^. E-shisha and other electronic nicotine delivery systems may conceivably be used by youth as an alternative means of accessing nicotine in situations where it may be cumbersome accessing the traditional shisha device, or among those who are otherwise concerned about the harmfulness of smoking traditional hookah^38^. More nuanced surveillance of emerging tobacco products is warranted, including reasons for use, brands, pricing and price minimizing strategies, terminology among youth, advertising exposure, and points of sale. A comprehensive approach to policy may be beneficial given the variability in the tobacco products on the African market. In domesticating the WHO Framework Convention on Tobacco control^39^, member states should consider their unique tobacco control landscapes and how tobacco control and prevention efforts could be strengthened using the different elements of MPOWER. Given that similar products can result in different population-level health outcomes based on the cultural context of their use in a region (and some variations in products as well), a domestic strategy to comprehensive tobacco prevention and control that considers local contextual factors is beneficial.

### Limitations

First, data on e-cigarettes and shisha were not available for all countries. Second, all measures were self-reported and may be subject to misclassification. Third, only associations can be inferred because of the cross-sectional design. In other words, while the article quantifies the gender difference in use of different tobacco products, it is not equipped to address what causes the gender differences in smoking among the surveyed youth. Fourth, the pooled multivariable analyses comprising the five countries with data for all four assessed products may not be representative of the African region. Fifth, due to the use of numerous comparisons, there is a chance that type 1 errors (false positives) could occur in the study. It is crucial to recognize that the chance of false positives still exists and should be taken into account when interpreting the data, even though the study addressed this issue by utilizing 99% confidence intervals and conservative assessments of adjusted prevalence ratios. Finally, GYTS is designed to be representative only of participants aged 13–15 years in the respective countries. Youth in older age groups might present different findings. Future research could explore gender differences in institutionalized youth or those dropping out of school, as they are more prone to engage in tobacco use.

## CONCLUSIONS

In many countries, boys reported a significantly higher prevalence of ever and current cigarette smoking than girls. However, among those who ever smoked cigarettes, a significantly higher percentage of girls reported an earlier age of initiation compared to boys. Furthermore, girls who currently smoked did not differ from their male counterparts in terms of smoking frequency or intensity. Also, use prevalence for other tobacco products was largely comparable between boys and girls. The identified prevalence and patterns of tobacco use in this young population afford opportunities for interceptive measures before they progress to full nicotine dependence. Introduction of plain packaging, complete bans on tobacco advertising, promotion, and sponsorships, restricting sales of cigarettes in single sticks, increasing tobacco taxes, and revamping education on the harmfulness of tobacco use are evidence-based measures that can help reduce youth tobacco use.

## Data Availability

Data sharing is not applicable to this article as no new data were created.
