# Supplementary material for: Gender differences in use of cigarette and non-cigarette tobacco products among adolescents aged 13–15 years in 20 African countries
Source: Tob Induc Dis. 2024 Jan 22;22:10.18332/tid/169753. doi: 10.18332/tid/169753 (PMC10801700; doi:10.18332/tid/169753)
Supplement: Supplementary file 1 [file TID-22-20-s1.pdf]

Supplemental Figure. Different tobacco products assessed among surveys of students aged 13-15 years in 20 countries in the WHO Africa region, Global Youth Tobacco Survey, 2013-2020

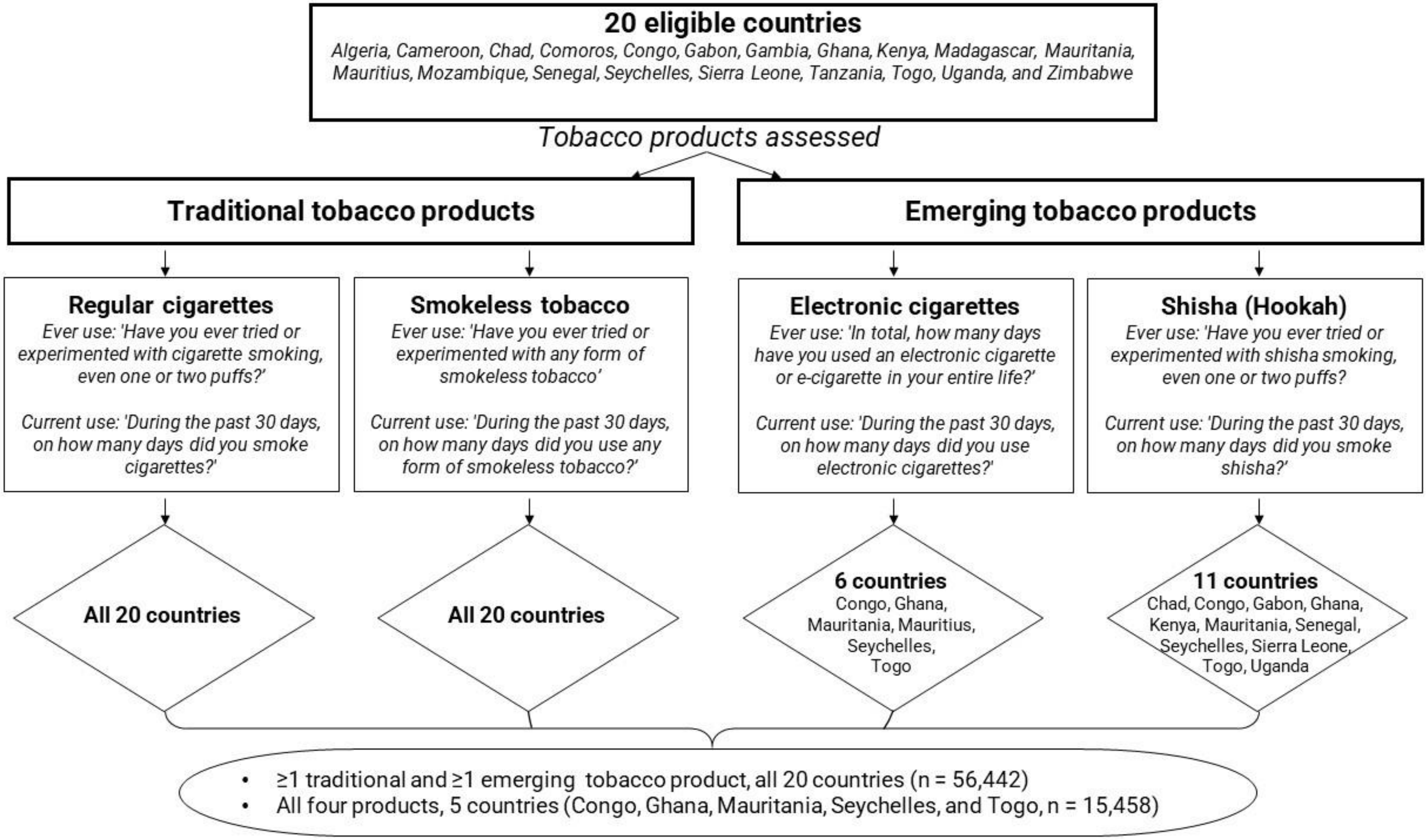

© 2024 Agaku I.T. et al.
